# Interaction between hippocampal-prefrontal plasticity and thalamic-prefrontal activity

**DOI:** 10.1038/s41598-018-19540-6

**Published:** 2018-01-22

**Authors:** Lezio S. Bueno-Junior, José E. Peixoto-Santos, Rafael N. Ruggiero, Milton A. V. Ávila, Danilo B. Marques, Cleiton Lopes-Aguiar, João P. Leite

**Affiliations:** 1 Department of Neurosciences and Behavioral Sciences, Ribeirão Preto Medical School, University of São Paulo, Ribeirão Preto, 14049-900 Brazil; 2 Núcleo de Neurociências, Department of Physiology and Biophysics, Institute of Biological Sciences, Federal University of Minas Gerais, Belo Horizonte, 31270-901 Brazil

## Abstract

The prefrontal cortex integrates a variety of cognition-related inputs, either unidirectional, e.g., from the hippocampal formation, or bidirectional, e.g., with the limbic thalamus. While the former is usually implicated in synaptic plasticity, the latter is better known for regulating ongoing activity. Interactions between these processes via prefrontal neurons are possibly important for linking mnemonic and executive functions. Our work further elucidates such dynamics using *in vivo* electrophysiology in rats. First, we report that electrical pulses into CA1/subiculum trigger late-onset (>400 ms) firing responses in the medial prefrontal cortex, which are increased after induction of long-term potentiation. Then, we show these responses to be attenuated by optogenetic control of the paraventricular/mediodorsal thalamic area. This suggests that recruitment and plasticity of the hippocampal-prefrontal pathway is partially related to the thalamic-prefrontal loop. When dysfunctional, this interaction may contribute to cognitive deficits, psychotic symptoms, and seizure generalization, which should motivate future studies combining behavioural paradigms and long-range circuit assessment.

## Introduction

Excitatory projections from the hippocampal CA1/subiculum area (CA1/sub) and the dorsal midline thalamus have overlapping terminal fields in the rat medial prefrontal cortex (mPFC), which in turn reciprocates its thalamic afferents^1–3^. Such a convergence occurs at the single-cell level, as a proportion of mPFC neurons are known to respond to orthodromic stimulation of both CA1/sub and limbic thalamus^4^, suggesting a hippocampal and thalamic cooperation within the mPFC. In fact, hippocampal and thalamic inputs to the mPFC have been demonstrated to gate each other, depending on co-stimulation parameters^5^. Furthermore, mPFC field responses to either hippocampal or thalamic pulses have been reported to undergo learning-associated synaptic plasticity, with each pathway showing a specific time course of plasticity over days of training^6^. Therefore, short-term cooperation and long-term plasticity across mPFC inputs might underlie the continuum between executive and mnemonic functions.

Thalamocortical loops sustain varying degrees of excitatory reverberation and network plasticity throughout states of vigilance^7^. These levels of excitability are currently proposed to regulate attention and working memory through the amplification of intra-cortical connectivity^8^, including in prefrontal cortical subsystems^9,10^. Consistently, cholinergically driven oscillatory states had been shown to modulate hippocampal-prefrontal^11,12^ and thalamic-prefrontal plasticity^13^. A more direct link between hippocampus-mPFC plasticity and thalamic activity comes from pharmacological inhibition studies^14–17^. Specifically, intra-thalamic lidocaine or MK-801 weakens CA1/sub-mPFC paired-pulse facilitation^14,15^, and intra-thalamic muscimol or tetrodotoxin reduces CA1/sub-mPFC single pulse recruitment^16,17^. Also of note, MK-801 into the midline thalamus, but not mPFC, reproduces the systemic effects of this drug on urethane-driven delta oscillations^15^, while muscimol or tetrodotoxin into the same thalamic area abbreviates subiculum-generated paroxysms^16,17^. Altogether, these findings support a common involvement of the thalamic-prefrontal loop and hippocampal-prefrontal plasticity in seizure spread and psychosis-relevant NMDA antagonism, as previously discussed^18^.

According to the studies above, the hippocampal-prefrontal plasticity may depend on the ongoing thalamic-prefrontal activity, which is still underexplored. Evaluating whether these processes control one another is important for understanding mPFC operations and their dysfunctions. Our two-experiment study further elucidates such relationship through electrophysiology, immunohistochemistry, and optogenetics. In chronically implanted rats, we observed that long-latency (>400 ms) mPFC firing responses to CA1/sub electrical pulses increase with hippocampal-prefrontal long-term potentiation (LTP). Then, we found that this >400 ms response is attenuated by PV/MD archaerhodopsin activation under anaesthesia. Thus, our data show the temporal profile with which the CA1/sub and limbic thalamus co-modulate the mPFC activity. These response patterns might inform future research with learning paradigms and animal models of psychiatric diseases.

## Results

### Design and sample sizes

**Figure 1:**
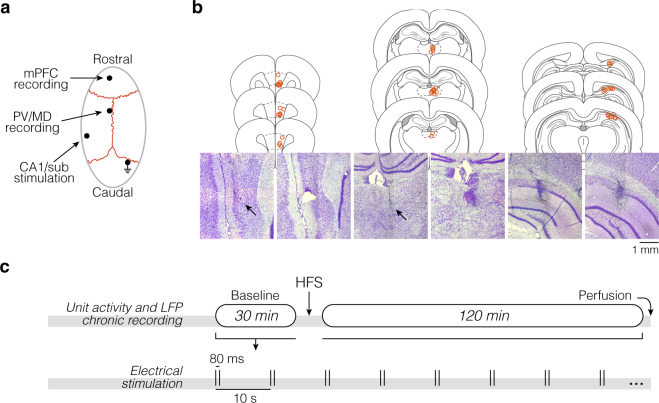
Electrode placements and experimental design. (**a**) Craniotomy sites in relation to skull sutures. Micro-screw holes are omitted, except for the one used as ground. Electrode connectors and microscrews were covered together with acrylic cement, aiming at chronic recordings. (**b**) Coronal sections and Nissl histology. Drawings were made based on the Paxinos and Watson rat brain atlas^19^. Red circles situate the electrolytic lesions across rats. Photomicrographs from two rats represent each brain site, with arrows indicating subtle lesions. (**c**) Timeline of the chronic recording session, undertaken once per rat. Electrical paired pulses (80 ms separation) were delivered into CA1/sub every 10 s throughout the session, except during HFS. Perfusion was made within 30 min after the recording session.

We monitored thalamic-prefrontal single-unit activity (SUA) and field postsynaptic potentials (fPSPs) while electrically stimulating CA1/sub in chronically implanted rats. Subjects received a stimulating electrode in CA1/sub, and recording microwires in mPFC and PV/MD (Fig. 1a,b). Then, in a single chronic session, the circuit was probed with electrical paired-pulse stimulation (0.1 Hz, 80 ms inter-pulse interval) before (30 min) and after (120 min) LTP induction with high-frequency stimulation (HFS; Fig. 1c). Therefore, subjects were their own controls. A ~15 min habituation preceded each session.

A total of 22 rats were used, five of which were lost because of inconsistent fPSP, or poor signal throughout channels. Thus, the main dataset is from 17 rats. Three of them presented poor signal in field potential channels specifically, and hence were excluded from fPSP analyses. Our SUA yield was of 44 mPFC units and 31 PV/MD units across rats. Apart from the 44 principal mPFC units, eight mPFC units fit the criteria of putative fast-spiking interneurons (FSI): >10 Hz spontaneous firing, <0.3 ms between valley and after-hyperpolarization peak^20^. Sample sizes will be referred to as N (rats) or n (units).

### mPFC, but not PV/MD, showed a late-onset HFS-sensitive response to CA1/sub pulses

**Figure 2:**
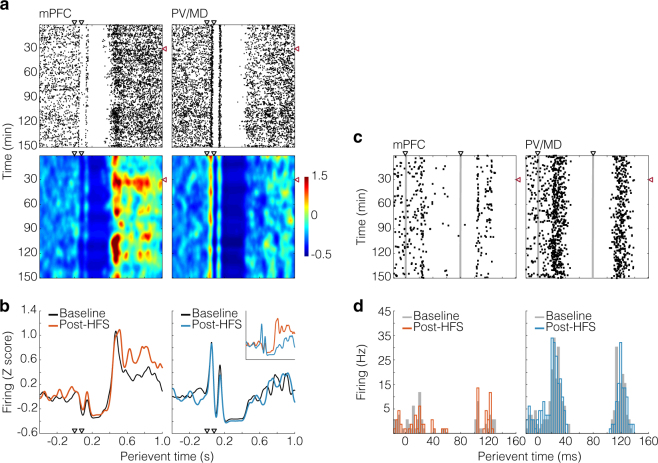
Representative prefrontal and thalamic units. (**a**) Top: 1.4 s-windowed raster plots (400 ms pre-event, see aligned to the x-axes of panel (**b**), showing spiking responses to CA1/sub paired pulses (y-axis: 180 baseline and 720 post-HFS sweeps, every 10 s). Bottom: color-coded arrays from the same raster plots. Spike counts (10 ms bins) were Z-scored against pre-event bins, and merged every 6 sweeps. Resulting arrays were then smoothed and plotted on a colour scale. Black and red arrowheads respectively indicate the paired pulses and HFS delivered into CA1/sub. (**b**) Perievent data from the smoothed raster plots of panel a. Sweeps were averaged from the final 15 min of the baseline, and initial 15 min of the post-HFS monitoring. Curves were then plotted on the Z-scored firing axis (y). The inset particularly compares post-HFS curves. (**c**) 180 ms-windowed raster plots from the same single units. Stimulus artefacts form grey vertical lines, indicating the timing between CA1/sub pulses and spiking responses. All other analyses are artefact-free. (**d**) Corresponding 3 ms-binned histograms.

Figure 2a,b depicts two representative neurons, one from each recorded site. The analysis includes perievent raster plots (Fig. 2a), and corresponding Z-scored smoothed histograms (Fig. 2b; 10 ms bins) comparing the baseline *vs*. the post-HFS period. According to Fig. 2a,b, both units showed paired pulse-locked excitatory responses (<200 ms latency), followed by a transient suppression (~200–400 ms), and then a new excitatory response (~400–800 ms). This secondary excitation was stronger in the mPFC unit, and unlike the PV/MD it was potentiated by HFS. A closer look at paired pulse-locked responses is provided by Fig. 2c (180 ms perievent window), illustrating how spikes (black dots) were timed in relation to stimulus artefacts (grey vertical lines). Corresponding histograms (Fig. 2d; 3 ms bins) show excitatory responses to each pulse (~20–40 ms latency), which were stronger in the thalamic unit, and indifferent to HFS in both units.

**Figure 3:**
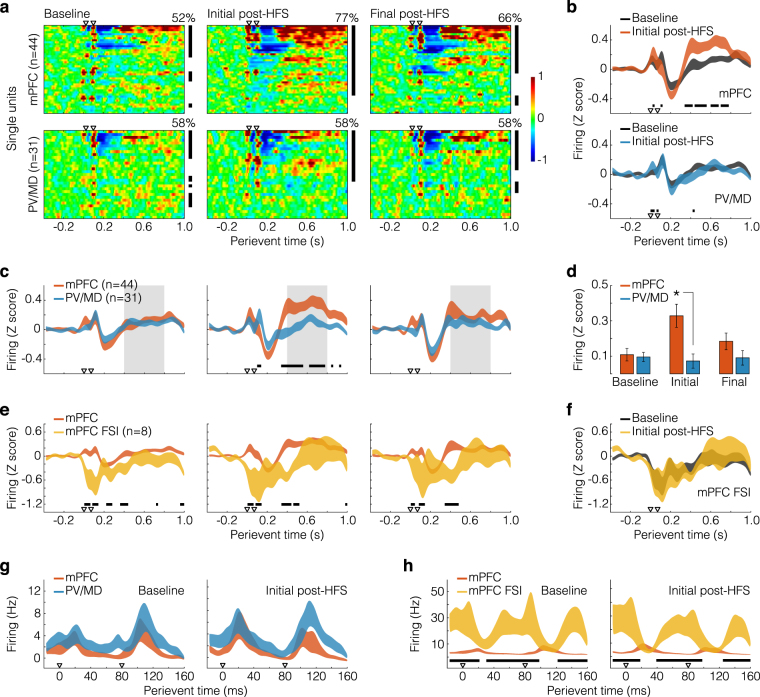
Distinct firing responses among samples of units. (**a**) Perievent histograms (400 ms pre-event, 10 ms bins) were Z-scored against pre-event bins, and averaged from the baseline (its final 15 min), initial post-HFS monitoring (15 min from HFS), and final post-HFS monitoring (last 15 min of the recording session). Histograms of individual units (rows of each array) were then sorted according to the mean moduli of post-event Z scores, specifically from the initial post-HFS period (i.e., the stronger the post-HFS response, the higher the row position). Resulting arrays were plotted on a colour scale. Arrowheads indicate CA1/sub paired pulses. Vertical bars on the right of each image indicate which single units were significantly responsive to CA1/sub paired pulses (pre- vs. post-event comparisons through t-tests). The moduli of 10 ms-binned Z scores were used in this particular analysis, thus capturing response magnitudes regardless of their directions (suppression or excitation). Proportions of mPFC responsive units significantly varied across periods, according to a chi-square test (see text). (**b**) Standard errors from the heatplots, after binning conversion (to 20 ms) and smoothing. The graphs compare basal and initial post-HFS data within recorded sites. Horizontal bars indicate Tukey’s *post-hoc* differences (*p* < 0.05) after two-way repeated measures ANOVA. (**c**) Same as in panel b, but comparing mPFC vs. PV/MD within recording periods. Grey areas indicate which time bins (0.4–0.8 s) were used for panel d analysis. (**d**) Focus on the late-onset excitation. Long-latency time bins (0.4–0.8 s) were averaged into a single Z score value for each unit. Means ± standard errors were then plotted as bars. The asterisk indicates a Tukey’s *post-hoc* difference (*p* < 0.05). (**e**) Same as in panel c, but comparing mPFC principal units and putative FSI. (**f**) Same as in panel b, but analysing putative FSI. (**g**) 180 ms-windowed rate histograms (3 ms bins) comparing mPFC and PV/MD within recording periods. (**h**) Same as in panel g, but comparing mPFC principal units and putative FSI.

Figure 3a–f depicts the samples of neurons using the 1.4 s perievent window. First, each heatplot of Fig. 3a shows a gradient of response intensity, ranging from no response to the representative patterns of Fig. 2a,b. As indicated by red areas, late-onset (>400 ms) excitatory responses to CA1/sub pulses were more prevalent among mPFC units, especially during the initial post-HFS period. In more details, the heatplots of Fig. 3a show averaged data from three periods: baseline (its second half), initial 15 min post-HFS, and final 15 min of the session. The rows of each heatplot are perievent histograms from individual units (except putative FSI), and columns are time bins (10 ms). Red, blue, and green tones respectively represent excitation, suppression, and no change (Z-scores against the 400 ms pre-pulse period). Of note, single units were sorted from top to bottom according to the mean moduli of post-pulse Z scores during the initial post-HFS period. Thus, the stronger the response within the 15 min after LTP induction, the higher the row position across images. As can also be seen, the transient suppression and the re-excitation varied in their durations and latencies (<200 ms variation), which can reflect uncontrolled microwire positioning in different mPFC layers (see Methods; Surgery and electrodes subsection), not to mention between-subject factors.

Black bars on the right of each heatplot (Fig. 3a) indicate which single units were responsive to CA1/sub paired pulses, irrespective of <200 ms variations. More specifically, we compared 10 ms-binned Z scores before (0.4 s) *vs*. after (1 s) pulses through t-tests, in a unit-wise manner. Z scores were converted to moduli in this particular analysis, thus capturing response magnitudes regardless of their directions. Figure 3a also shows the proportions of responsive units. According to a chi-square test, HFS modulated these proportions in the mPFC (*χ*^2^ = 6.073; *p* = 0.048), but not PV/MD.

Further comparisons between mPFC and PV/MD are represented by Fig. 3b–d. Data from each heatplot were converted to 20 ms-binned smoothed curves, with shaded areas delimiting standard errors. Figure 3b compares recording periods within brain sites, whereas Fig. 3c does the opposite. Figure 3b,c confirms that the HFS-sensitive late-onset excitation was stronger in mPFC, as attested by statistical outputs: Fig. 3b mPFC (baseline *vs*. initial post-HFS: F_(1,4214)_ = 4.561, *p* = 0.036; interaction: F_(49,4214)_ = 2.970, *p* < 1 × 10^–5^), Fig. 3b PV/MD (interaction: F_(49,2940)_ = 1.579, *p* = 0.006), and, noteworthy, Fig. 3c initial post-HFS (mPFC *vs*. PV/MD: F_(1,3577)_ = 5.635, *p* = 0.020; interaction: F_(49,3577)_ = 3.660, *p* < 1 × 10^−5^). Statistical effects of perievent time were detected in all five datasets: Fig. 3b (mPFC: F_(49,4214)_ = 17.578, *p* < 1 × 10^−5^; PV/MD: F_(49,2940)_ = 11.937, *p* < 1 × 10^−5^), and Fig. 3c (baseline: F_(49,3577)_ = 12.370, *p* < 1 × 10^−5^; initial post-HFS: F_(49,3577)_ = 13.274, *p* < 1 × 10^−5^; final post-HFS: F_(49,3577)_ = 14.560, *p* < 1 × 10^−5^). Figure 3d further supports these observations. First, the 400–800 ms post-pulse bins (grey areas of Fig. 3c) were averaged into a single Z score value unit wise. These values were then plotted as the mean ± standard errors of Fig. 3d, highlighting the difference between mPFC and PV/MD during the initial post-HFS monitoring (recording periods: F_(2,146)_ = 7.464, *p* = 8.2 × 10^−4^; interaction: F_(2,146)_ = 11.476, *p* = 2.3 × 10^−5^).

In summary, these findings reveal that responses to CA1/sub paired pulses varied between mPFC and PV/MD, especially at the 400–800 ms latency (Fig. 3c grey areas), during which the mPFC excitation was transiently potentiated by HFS.

### mPFC putative FSI and principal cells responded in opposite manners to CA1/sub pulses

Figure 3e replicates the mPFC curves of Fig. 3c, this time to compare between mPFC principal cells and putative FSI. According to Fig. 3e, the activity of putative FSI tended to be decreased (<160 ms latency), increased (~160–300 ms), and decreased again (~300–500 ms), as opposed to the sequence of phasic excitation, transient suppression, and re-excitation of principal cells (mPFC *vs*. FSI: Fs_(1,2450)_ ≥ 7.558, *p*s ≤ 8 × 10^−3^; effects of perievent time: Fs_(49,2450)_ ≥ 3.578, *p*s < 1 × 10^−5^; interaction: Fs_(49,2450)_ ≥ 2.961, *p*s < 1 × 10^−5^). Figure 3f, in turn, corresponds to no statistical effects other than perievent time (F_(49,686)_ = 3.733, *p* < 1 × 10^−5^), meaning that HFS did not change the response of putative FSI to CA1/sub pulses.

### 180 ms-windowed responses of the three samples of units to CA1/sub pulses

Figure 3g provides a closer look at the immediate responses to CA1/sub stimuli (180 ms perievent, 3 ms bins), similarly to Fig. 2d. Only effects of perievent time were found (baseline: F_(59,4307)_ = 7.639, *p* < 1 × 10^−5^; initial post-HFS: F_(59,4307)_ = 7.424, *p* < 1 × 10^−5^), meaning that mPFC and PV/MD responses were similar between each other and indifferent to HFS. With this we can conclude that HFS-induced changes in evoked firing were restricted to the mPFC late-onset excitation. Also within the 180 ms timeframe, Fig. 3h compares mPFC principal cells and putative FSI, reinforcing the inverse relationship between their response profiles (mPFC *vs*. FSI: Fs_(1,2950)_ ≥ 38.184, *p*s < 1 × 10^−5^; effects of perievent time: Fs_(59,2950)_ ≥ 6.204, *p*s < 1 × 10^−5^; interaction: Fs_(59,2950)_ = 9.928, *p*s < 1 × 10^−5^).

### Plasticity of fPSP responses and spontaneous firing rates

**Figure 4:**
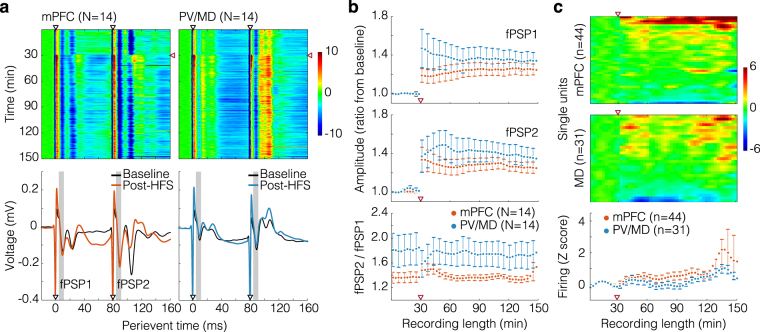
Long-term increase of field responses and spontaneous firing induced by HFS. (**a**) Top: fPSP sweeps (180 basal, 720 post-HFS, every 10 s) were averaged across rats, and stacked within the 180 ms perievent window (20 ms pre-event, see aligned to the x-axes of the bottom graphs). Each sweep was then Z-scored against its own pre-event segment, and the resulting arrays were plotted on a colour scale. Black and red arrowheads respectively indicate the paired pulses and HFS delivered into CA1/sub. Bottom: fPSP were averaged from the final 15 min of the baseline, and initial 15 min of the post-HFS monitoring, also across rats. Resulting curves were plotted on the voltage axis (y). Grey areas indicate the latency windows (5–14 ms) used for panel b analyses. (**b**) LTP of fPSP1 and fPSP2 (top and centre) and paired-pulse ratio (bottom) in 3 min blocks (i.e., every 18 fPSP) shown as mean ± standard errors. LTP data are ratios from the baseline mean amplitude, and red arrowheads indicate the CA1/sub HFS. (**c**) Top: 3 min-binned spike count histograms were Z-scored against the baseline, and stacked into numerical arrays (see aligned to the x-axis of the bottom graph). Histograms were then sorted in descending order of the mean post-HFS Z score, and plotted on a colour scale. Bottom: mean ± standard errors from mPFC and PV/MD histograms.

Figure 4a depicts fPSPs in 180 ms perievent windows. The heatplots (top panel) show Z-scored fPSP sweeps throughout the recording timeline, after averaging across rats. In turn, the waveforms (bottom panel) show averaged fPSP from the baseline and initial post-HFS period. Both mPFC and PV/MD manifested short- and long-latency valleys (respectively 10–12 and 21–23 ms from each pulse), although the second valley was more evident in the mPFC (W-shaped pattern), while in PV/MD it appeared like a subcomponent of a rather positive deflection (Fig. 4a).

Presumptively monosynaptic fPSP components (5–14 ms latency; Fig. 4a, bottom panel; grey areas)^1,16,17,21,22^ were used for the analyses of Fig. 4b. Amplitudes were initially averaged in 3 min blocks. Then, for generating the top and middle graphs of Fig. 4b, mean ± standard errors were plotted as ratios from the baseline mean. Through analysing the entire recording length, we found only effects of time (fPSP1: F_(49,1274)_ = 11.853, *p* < 1 × 10^−5^; fPSP2: F_(49,1274)_ = 11.710, *p* < 1 × 10^−5^). No statistical effects were observed when analysing the post-HFS period. Thus, the 2 h post-HFS monitoring showed a constant LTP in both mPFC and PV/MD. In turn, fPSP2/fPSP1 amplitude ratios did not significantly differ between mPFC and PV/MD (mPFC *vs*. PV/MD: F_(1,274)_ = 2.885, *p* = 0.101; Fig. 4b, bottom panel).

Figure 4c describes the spontaneous firing rates of mPFC and PV/MD neurons (heatplots), and their mean ± standard errors (bottom graph) in 3 min bins. Heatplot rows consist of individual rate histograms, which were Z-scored against the baseline, smoothed, and sorted in descending order of the mean post-HFS Z score. This shows the diversity of SUA throughout the recording. Results show that a cumulative excitation began to emerge ~90 min after HFS (Fig. 4c). This net change was confirmed by effects of time (with baseline: F_(49,3577)_ = 3.402, *p* < 1 × 10^−5^; without baseline: F_(39,2847)_ = 2.982, *p* < 1 × 10^−5^). No differences were found between mPFC and PV/MD.

Thus, both recorded areas underwent long-term changes after CA1/sub HFS: potentiation of field responses and net excitation of spontaneous firing. This contrasts with the evoked firing data (Fig. 3b–d), whose changes were confined to the mPFC and the initial post-HFS period. More generally, the effects of HFS showed distinct time courses depending on which dimension of neural activity was analysed: either evoked firing, or LTP and spontaneous firing.

### Correlation between Zif268 expression and electrical recruitment

**Figure 5:**
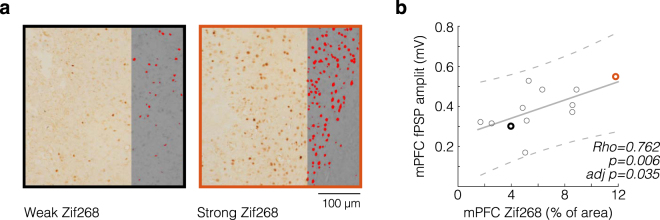
Correlation between mPFC immediate early gene expression and fPSP amplitude. (**a**) Representative mPFC specimens with weak (black frame) and strong (orange frame) Zif268 expression, each from a rat. Photomicrographs are partially covered with their 8 bit-greyed versions, whose red masked areas indicate Zif268 immunopositivity by means of a threshold tool (ImageJ). (**b**) Scatter plot of prefrontal Zif268-positive areas (x-axis) against fPSP2 amplitudes (y-axis) from multiple rats (circles). Two of 14 mPFC coronal sections were lost, resulting in the 12 circles of the scatter plot. Highlighted circles correspond to the specimens of panel a. The positive correlation (Spearman’s, with False Discovery Rate adjustment) is depicted by a polynomial fit (solid line) and 95% confidence bounds (dashed lines).

Immediate early genes are activated upon neural stimulation^23^. In particular, Zif268 expression is associated with NMDA receptor-mediated transmission, and synaptic plasticity^24,25^. The objective, here, was to examine Zif268 expression in relation to mPFC and PV/MD response evoking. Figure 5 shows a significant Spearman’s correlation between prefrontal Zif268 immunopositivity and prefrontal fPSP amplitude, after a False Discovery Rate adjustment (*Rho* = 0.762, *p* = 0.006, adjusted *p* = 0.035). Thus, the stronger the prefrontal fPSP response to CA1/sub pulses (specifically after HFS), the stronger the prefrontal Zif268 expression (Fig. 5). Correlations were consistently absent when analysing thalamic fPSP, thalamic firing, and prefrontal firing. Therefore, we found a relationship between Zif268 expression and the CA1/sub-mPFC recruitment. These data show that stimulation effectiveness was commensurate with a non-electrophysiological assessment of the circuit.

### Complementary optogenetics

#### Purpose and design

**Figure 6:**
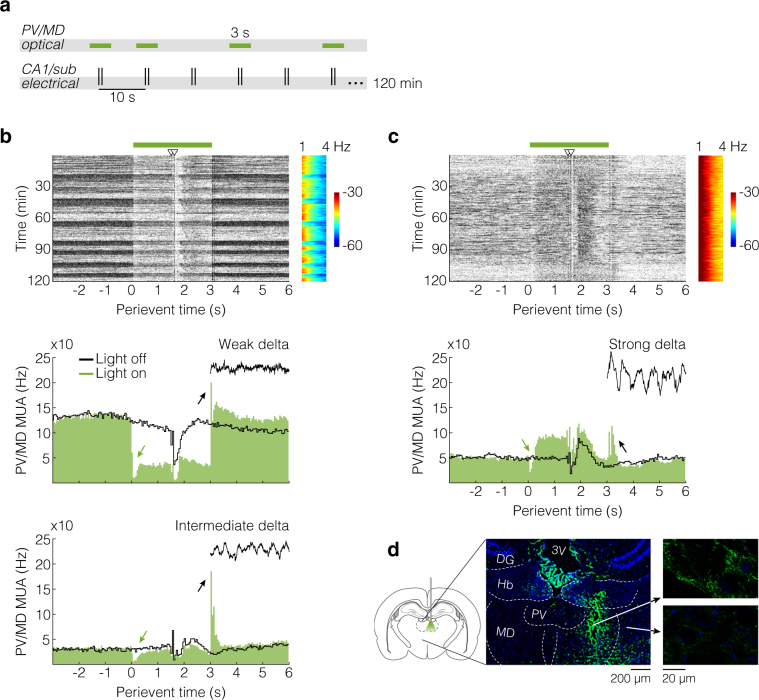
Complementary co-stimulation experiment: thalamic optical drive under urethane anaesthesia. (**a**) Experimental design. Electrical paired pulses were delivered into CA1/sub every 10 s for 120 min, either with or without 3 s light pulses into PV/MD, randomly. (**b**) Rat 1 data. Top: perievent raster plot describing MUA reactions of PV/MD to light pulses (green bar) and CA1/sub electrical paired pulses (arrowheads). The raster plot is aligned to an LFP spectrogram (shared y-axis) demonstrating the spontaneous cycling between “activated” and “deactivated” oscillatory states^27^ (here called weak and intermediate delta, respectively; colour scale: decibels). These oscillatory states correspond to the horizontal bands of the raster plot, whose sweeps were analysed through separate histograms (bottom graphs). Light-onset suppressions (<250 ms duration) and light-offset rebounds (~50–250 ms latency) are indicated (green and black arrows, respectively). (**c**) Rat 2 data. Top: same organization of panel b. In this case we observed a state of persistently strong delta oscillations. Hence, all data were analysed as a single histogram (bottom). (**d**) Left: optrode positioning, illustrating the penetration of the green light cone (525 nm wavelength). Drawings were made based on the Paxinos and Watson rat brain atlas^19^. Centre: coronal section demonstrating the plasma membrane expression of archaerhodopsins, as evidenced by eYFP fluorescence (green tones). Right: closer views of strongly (top) and weakly (bottom) eYFP-marked cells depending on the distance from the injection/stimulation site. Cell nuclei were stained with Hoechst 33342 (blue tones). 3 V, third ventricle; DG, dentate gyrus; Hb, habenula.

As a follow-up to the main experiment, we explored the thalamic role in the CA1/sub-mPFC recruitment. Rats were transfected with AAV5-hSyn-eArch3.0-eYFP in PV/MD for expression of archaerhodopsins (green-light gated outward proton pumps^26^). A month later, rats were anesthetized with urethane and implanted as above, except for an optrode into PV/MD. This time, LTP was not induced, so we could focus on basal CA1/sub-mPFC-PV/MD interactions using optical and electrical co-stimulation (Fig. 6a). Briefly, the CA1/sub was electrically stimulated (every 10 s, 120 min) while recording from mPFC and PV/MD, similarly to the main experiment. This time, PV/MD optical pulses (3 s) randomly accompanied the CA1/sub pulses at the probability of 50%, and no HFS was applied. Data are from two rats, which represent the most successful recordings out of six attempts (see Methods for exclusion criteria).

#### Validation

We initially report data from PV/MD, specifically. The raster plot of Fig. 6b shows unsorted multi-unit activity (MUA) from rat 1. Light pulses elicited a sustained inhibition in PV/MD, only disrupted by CA1/sub stimuli, as demonstrated by the central area of the raster plot. Also noticeable are the horizontal bands of the raster plot (Fig. 6b). They represent sweeps with low (light tones) or high (dark tones) thalamic firing rates, which coincide with changes in thalamic local field potentials (LFP), as shown by the aligned spectrogram (shared y-axis). These LFP changes reflect the sleep-like alternation between “activated” and “deactivated” patterns of urethane anaesthesia, as previously described based on delta oscillations^27^. Thalamic firing modes are crucially involved in these oscillatory patterns^28^, and therefore we decided to sort the sweeps based on their basal rates. In other words, each sweep was assigned a firing rate based on the 3 s period before light. Rates were then categorized using percentiles: high rates (above 55th) and low rates (below 45th), thus ruling out dubious transition states^27^.

Sweep categorization resulted in the histograms of Fig. 6b (50 ms bins). Respectively, top and bottom histograms correspond to “activated” states (here called weak delta) and “deactivated” states (here called intermediate delta). As indicated by arrows, we observed transient suppressions at light onset (<250 ms duration), and thalamic rebounds^29^ at light offset (~50–250 ms latency), regardless of the oscillatory state. The sustained inhibition, however, was specific to the weak delta background. Responses to CA1/sub pulses were similar to those of the main experiment, and were consistently observed across trials and oscillatory states (Fig. 6b). In turn, Fig. 6c shows PV/MD data from rat 2, which manifested a state of persistently strong delta oscillations. Light-onset suppressions (green arrow) and light-offset rebounds (black arrow) were again observed. In this rat, however, light pulses evoked a plateau excitatory response, only disrupted by CA1/sub stimuli (Fig. 6c). Figure 6d finally exemplifies the fluorescence-evidenced expression of archaerhodopsins in the optrode-implanted area (green tones). Blue tones correspond to Hoechst-stained nuclei.

#### Disruption of CA1/sub-mPFC responses by thalamic optical drive

**Figure 7:**
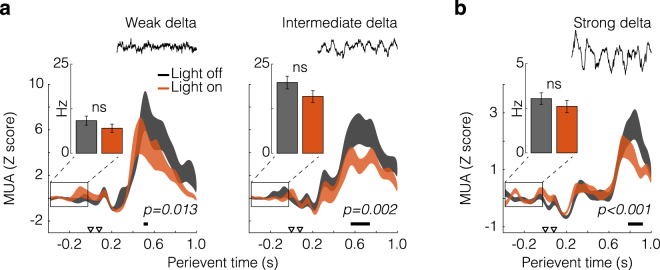
Complementary co-stimulation experiment: effects of thalamic optical drive on CA1/sub-mPFC responses. (**a**) Rat 1 data analysed as in Fig. 3b,c (400 ms pre-event, 20 ms bins), with standard errors representing trial variability (i.e., subject as its own control). Sweeps occurring in different oscillatory states were separately analysed (see also Fig. 6b). (**b**) Rat 2 data (see also Fig. 6c). Arrowheads indicate the paired pulses delivered into CA1/sub. Horizontal bars indicate Tukey’s *post-hoc* differences (*p* < 0.05) after two-way repeated measures ANOVA. Insets show mPFC firing in the 400 ms period before CA1 stimuli. Inset data were not Z scored, thus clarifying whether PV/MD light pulses were affecting the mPFC activity immediately prior to CA1 stimuli. No significant differences were found (ns), which further validates the Z scored data.

Figure 7 represents the main finding of this complementary experiment. It basically shows that PV/MD optical drive affects CA1/sub-driven activity in the mPFC, regardless of the activity background. Specifically, Fig. 7 depicts Z-scored 1.4 s-windowed data from mPFC, similarly to previous analyses (Fig. 3b,c), except that standard errors now reflect trial variability, i.e., subjects are their own controls. Figure 7a data are from rat 1, which manifested the spontaneous sleep-like cycling of oscillatory states. As can be seen, PV/MD light pulses were able to attenuate the >400 ms excitation in both activity states: weak delta (effect of perievent time: F_(49,2254)_ = 21.794, *p* < 1 × 10^−5^; interaction: F_(49,2254)_ = 1.510, *p* = 0.013), and intermediate delta (effect of perievent time: F_(49,2254)_ = 31.215, *p* < 1 × 10^−5^; interaction: F_(49,2254)_ = 1.719, *p* = 0.002). Data from rat 2 corroborate this finding. As shown by Fig. 7b, the late-onset CA1/sub-mPFC response was again attenuated during light-on trials (effect of perievent time: F_(49,3136)_ = 32.350, *p* < 1 × 10^−5^; interaction: F_(49,3136)_ = 2.424, *p* < 1 × 10^−5^). Between-subject variations in long-latency responses can also be seen - i.e., rat 1 responses approximating the average pattern of the main experiment (Fig. 3b,c), and rat 2 responses starting ~200 ms later - consistently with the variability we reported earlier (Fig. 3a). Lastly, the insets show mPFC firing rates in the 400 ms period before electrical pulses in CA1, comparing light-on and light-off trials. Data were not Z scored in this analysis, so we could evaluate whether PV/MD light pulses were globally affecting the mPFC activity immediately prior to CA1 stimuli. According to t-tests, there were no significant differences (weak delta: t_(294)_ = 1.176, *p* = 0.241; intermediate delta: t_(294)_ = 1.544, *p* = 0.124; strong delta: t_(658)_ = 0.893, *p* = 0.372). This reinforces the effects shown in the main graphs of Fig. 7, i.e., >400 ms Z-scored firing.

Altogether, our two experiments demonstrate that late-onset mPFC responses are potentiated by HFS of CA1/sub, and attenuated by archaerhodopsin activation in PV/MD. This suggests a mutual relationship between hippocampal-prefrontal plasticity and thalamic-prefrontal activity, thus contributing to an emerging debate on intra-PFC amplification^9,10^, and long-range circuit interactions^30,31^. Future co-stimulation experiments with greater samples should expand on these observations.

## Discussion

Exogenously induced synaptic plasticity has many more consequences than just the changes in afferent responding. There is probably a myriad of polysynaptic events between short-latency pathway recruitments and their brain-wide outcomes. Systems-level attempts like the present one are useful to map these intermediate events. In our study, they seem represented by the long-latency prefrontal responses to hippocampal electrical pulses. Because these responses were sensitive to both hippocampus-induced LTP and thalamic optogenetic control, they might consist of a network plasticity mechanism, which could partially underlie the executive functions, in line with recent evidences^9,10^. Similar mechanisms may also occur in other thalamocortical subsystems, like those involved in motor and sensory operations^32^.

A speculation is that the CA1/sub-mPFC recruitment could either: (1) enable a longer-lived resonance within the thalamic-prefrontal loop (~400 ms latency and duration); (2) be, on the contrary, regulated by thalamic activity; or (3) combine both. In any event, the local mPFC circuit would undergo temporary states of increased or decreased net excitation. For example, at transiently enhanced depolarization, CA1/sub-innervated pyramidal cells of the mPFC would be more prone to respond to other afferents, e.g., from basolateral amygdala^33^. In this case, CA1/sub-mPFC-PV/MD interactions could provide time frames of increased limbic connectivity. These depolarization events could also foster NMDA receptor-dependent plasticity processes, e.g., cytosolic Ca^2+^-mediated AMPA receptor trafficking/anchoring, and immediate early gene activation^23,34^, transforming millisecond-range input convergence into lasting modifications of mPFC synaptic efficacy. In addition, the plastic changes we observed followed different time courses depending on which aspect of neural activity was analysed: either perievent firing (changes were stronger in the initial post-HFS period), 3 min-binned firing (changes were stronger in the final post-HFS period), or fPSP amplitudes (LTP was rather constant). As recently discussed^35^, early and late LTP reflect a temporal sequence of biochemical mechanisms. They possibly range from ionic conductance changes in AMPA receptors that are already available in the postsynaptic density (seconds to minutes), to increases in synaptic size and quantal transmission based on neuromodulator-mediated protein synthesis (hours)^35^. Future studies should dissect LTP-like firing patterns - like the ones described here - in light of these cellular and neurochemical mechanisms.

In a broader neurophysiological sense, the afferent cooperation within the mPFC is possibly modulated by the oscillatory state. We have previously reported that the same activity background - with either urethane-driven slow or cholinergically induced rapid oscillations - differentially modulates the mPFC plasticity depending on the stimulated site: either CA1/sub^11,12^ or PV/MD^13^. Further interpretation can be made based on the present data. Our recordings during sleep-like cycling indicated that optical pulses required rapid oscillations to induce sustained thalamic inhibition. This effect was indeed expected to be limited during slow oscillations, which reflect endogenous thalamic hyperpolarization^28^. The long-latency CA1/sub-mPFC response, on the other hand, was attenuated across oscillatory states. Intriguingly, this attenuation was replicated during strong delta oscillations, when light pulses paradoxically evoked plateau excitations. This suggests a partial dissociation between ongoing background activity (noise) and ephemeral polysynaptic interactions (signal), at least with respect to the exogenously manipulated CA1/sub-mPFC-PV/MD circuit. Thus, the balance between noise (ongoing thalamic activity) and signal (CA1/sub-evoked patterns) might fluctuate with the oscillatory context. It is anyway noticeable that the optical drive of PV/MD was unable to completely block long-latency mPFC responses, implying other subcortical resonators. Hence, we can also speculate that CA1/sub-mPFC information reverberates in multiple excitatory loops (e.g., between mPFC and ventral midline thalamus^36^), and that preferentially resonating in this or that loop could depend on the oscillatory activity.

Collaterally to the attenuation of late-onset mPFC responses, we observed different PV/MD reactions to archaerhodopsin activation, similarly to a previous report on the monkey visual cortex^37^. Specifically, we found a consistent <250 ms activity drop at light onset. This transient event was then followed by plateaus of either strong suppression, weak suppression, or excitation, in an apparent relationship with weak, intermediate, and strong delta power, respectively. This could suggest that oscillatory states may determine the direction of green light-driven PV/MD responses. If this is the case, PV/MD responses could reflect distinct combinations between the direct hyperpolarization of thalamocortical cells, and their disinhibition via GABAergic terminals from the reticular thalamus, both of which supposedly transfected with eArch3.0. Reticular-mediated disinhibition is indeed critical for translating neurochemical states (e.g., cholinergic tone) into global rhythms^38,39^, which could explain the relationship we found between delta power and the direction of PV/MD responses. However, we also recognize that plateau responses could reflect biophysical constraints of sustained archaerhodopsin activation, including pH-dependent presynaptic calcium influx and the consequent increase in local neurotransmitter release^40^. These issues should motivate further evaluation of PV/MD activity with different optogenetic tools and during different oscillatory backgrounds.

Apart from this discussion on long-latency thalamic-prefrontal interactions, we also found shorter-latency mPFC responses to CA1/sub stimuli: paired pulse-locked sharp increases (<50 ms), and ensuing inhibitory components (~160–400 ms). This post-stimulus pattern is in agreement with a previous investigation^4^. Using pharmacological tools, the authors have attributed the sharp excitatory events to AMPA receptor-mediated neurotransmission, and the inhibitory components to GABAergic interneuronal processing. Our putative FSI data, together with previous reports^41,42^, corroborate this interneuronal participation, since perievent patterns from putative FSI and the main sample of mPFC units were opposite. Similar sequences of phasic excitation then inhibition possibly exist throughout the neocortex, with each neocortical area employing such pattern to a specific function. At least in the mPFC, the excitation-inhibition sequence is likely to organize its receptivity and refractoriness to limbic inputs, including thalamic ones^5,43–45^.

Altogether, initial excitation, ensuing inhibition, and the slow re-excitation represent the temporal profile with which a proportion of mPFC cells react to CA1/sub stimuli. In addition, our work describes PV/MD reactions to CA1/sub pulses. As there are no known connections between CA1 pyramidal cells and the dorsal midline thalamus in rodents, the subicular recruitment could have been responsible for the PV/MD responses we observed^16,17,46–48^. CA1/sub is commonly approached as an integrated source of hippocampal formation outputs^49^. Thus, nonspecific electrical stimulation of such area could explain the thalamic fPSP we observed, in addition to the proportion (>50%) of responsive PV/MD units. That being said, these PV/MD responses may reflect a thalamus-relayed pathway from hippocampal formation outputs to the mPFC, similarly to the circuit comprising CA1, mPFC, and the thalamic reuniens and rhomboid nuclei^33^. Other trans-thalamic routes are indeed found across the brain^32^. They contribute to efference copies, which participate in the self-monitoring of actions^50,51^, and are disturbed in schizophrenia^52^. Given the associative mPFC role, its connectivity with the CA1/sub and PV/MD areas could be important for the cognitive self-monitoring. Further investigation on the PV/MD recruitment - e.g., using different paired-pulse protocols, or optical (rather than electrical) stimulation of CA1 or sub - could clarify these issues.

The limbic thalamic involvement in psychosis is evidenced by its response to locally infused MK-801 (non-competitive NMDA antagonist), which by itself replicates the drug’s systemic effects on both spontaneous and subiculum-evoked mPFC activity^15^. Together with the *post-mortem* evidence of thalamic cell loss in schizophrenia^53^, this reinforces that the mPFC undergoes thalamus-related downstream alterations in psychosis. The midline thalamus is also increasingly implicated in the spread of limbic seizures^54^. Pharmacological inhibition of the MD has been shown to abbreviate subiculum-generated after-discharges in the mPFC, in addition to attenuating its subiculum-evoked responses^16,17^. Considering the thalamic participation in temporal lobe epilepsy^55,56^, the PV/MD might be important for the comorbidity between this neurological disease and psychotic symptoms^18^, a research topic that could benefit from approaches like those reported here.

In summary, this study depicts sub-second-range prefrontal cortical and midline thalamic responses upon electrical stimulation of the hippocampal formation *in vivo*. Specifically in the prefrontal cortex, we observed a secondary excitatory response that was potentiated after hippocampal HFS, and attenuated during thalamic optical drive. Thus, our findings further describe how hippocampal-prefrontal-thalamic interactions are timed, including interneuronal processing within the prefrontal cortex. Although exogenously induced, these patterns give further idea of what occurs in limbic circuits during cognitive operations and their dysfunctions. Based on our contribution, future studies combining behavioural monitoring, multiple-site recording, and co-stimulation designs may advance the dissection of prefrontal-mediated flows of information.

## Methods

### Subjects

Adult male Wistar rats were housed in bedded cages under 12 h light/dark cycle (lights on at 7 AM), with food/water *ad lib* and standard temperature. Procedures followed the Brazilian Council for Control of Animal Experimentation guidelines. The local bioethics committee (Ribeirão Preto School of Medicine) approved our procedures (128/2014).

### Surgery and electrodes

Chronic implants were made under ketamine/xylazine anaesthesia. The craniotomy (burr holes) aimed at three bregma-referenced ipsilateral targets: mPFC (3.1 mm anterior, 0.5 mm lateral, 3.2 ventral from dura), PV/MD (1.9 mm posterior, 0.3 mm lateral, 5.1 mm ventral), and temporal CA1/sub (5.7 mm posterior, 4.5 mm lateral, ~2.5 mm ventral)^19^. See Fig. 1a,b.

Both the mPFC and PV/MD received handmade eight-channel microwire bundles for recording (teflon-coated tungsten, 50 μm). These probes allow multichannel recordings with simplicity of electrode making, but at the expense of lower anatomical precision (precluding the ability to assign channels to cortical layers, for example). In turn, the CA1/sub area received a bipolar electrode for monophasic stimulation (teflon-coated tungsten, 60 μm, ~500 μm inter-pole). For optimizing the CA1/sub coordinate, paired pulses were delivered through the bipolar electrode during its dorsal-ventral trajectory until fPSPs were consistently evoked.

Six microscrews, including a ground reference on the contralateral cerebellum, were fastened to the bone around the electrodes. The resulting miniature system was enclosed together on the skull with dental cement. Rats were then allowed to recover for 5–7 days.

### Recording and electrical stimulation

For surgery recordings, mPFC and PV/MD electrodes were connected to an analog-digital converter (ADInstruments) via a battery-operated preamplifier, aiming at fPSP acquisition (1 kHz low-pass, 1000x gain, 2 kHz digitization) upon CA1/sub stimulation. Stimuli consisted of pairs of square pulses (200 μs, 300 μA, 80 ms interpulse interval) delivered every 10 s. Pulses were generated from a stimulator and photoelectrically isolated (Grass Technologies). This system was merely used for optimizing the CA1/sub dorsal-ventral coordinate, as explained above. Reasons for probing the circuit with paired pulses were: (1) to ensure efficient afferent stimulation without increasing the current intensity; and (2) to differentiate between paired pulse-locked responses (dozens of milliseconds) and longer-latency responses.

For chronic sessions, rats were plugged into the stimulation/recording cables (including unity-gain headstages), and allowed to move freely in a soundproof box. Cables were connected to their devices (photoelectric isolator and preamplifier) on the outside of the box without using a commutator relay. We employed a multichannel acquisition processor (Plexon) with the following parameters. LFP: 0.7–500 Hz band-pass, 1000x gain, and 2 kHz digitization. MUA: 250–8000 Hz band-pass, 1000x gain, and 40 kHz digitization. CA1/sub stimulation was made as above (Fig. 1c), with intensity based on an input-output curve prior to recording (~200–400 μA, i.e., ~70% of maximum fPSP amplitude). LTP was induced by CA1/sub HFS: two series (10 min apart) of 10 trains (every 10 s), each train with 50 pulses at 250 Hz^11–13^.

For each paired pulse, a timestamp from the stimulator was sent to the multichannel system at 40 kHz digitization, allowing perievent analysis. Pulse artefacts expectedly contaminated the spike signal, but they could be removed through principal component analysis. Only then we proceeded to semiautomatic spike sorting, followed by clustering assessment through multivariate ANOVA (Offline Sorter), and elimination of redundant channels through crosscorrelation. Spike trains were finally saved as SUA data.

### Histology and immunohistochemistry

Within 30 min after the experiment, rats were perfused with phosphate-buffered saline (PBS) then 4% paraformaldehyde in PBS (200 mL each), and decapitated. Electrode tips were marked with electrolytic lesions (0.8 mA, 0.8 s). Brains were removed from skull, post-fixed in 4% paraformaldehyde, and embedded in paraffin. By making lesions after perfusion, we could examine the mPFC, PV/MD, and CA1/sub for Zif268 immunohistochemistry upon coronal sectioning (8 μm) and bright-field microscopy. Separate coronal sections were Nissl-stained to check electrode positioning.

Published immunohistochemical protocols were used^57^. Briefly, endogenous peroxidase was blocked with hydrogen peroxide in PBS (pH 7.4), followed by microwave antigenic retrieval in sodium citrate buffer (pH 6.0). Coronal sections were then incubated overnight in blocking buffer containing the polyclonal antibody against Zif268/Egr-1 (sc-189, Santa Cruz Biotechnology; 1:100 dilution). Primary antibodies were detected with the biotinylated anti-rabbit IgG (E0353, Dako; 1:100 dilution) followed by the HRP Kit (PK6100, Vector Laboratories), and finally revealed with diaminobenzidine.

### Optogenetics

Ketamine/xylazine-anesthetized rats were transfected with the AAV5-hSyn-eArch3.0-eYFP vector (10^12^ viral particles/mL in PBS; University of North Carolina Vector Core) into PV/MD using an infusion pump-driven microsyringe. The infusion rate was 0.1 μL/min (0.3 μL total). The aim was to induce the PV/MD expression of archaerhodopsins (green light-gated outward proton pumps^26^). Four weeks were allowed for archaerhodopsin expression^58^, after which we performed recordings under urethane anaesthesia. Implants were the same as the main experiment, except for a handmade optrode into PV/MD. Only the mPFC microwire bundle was fixed in place with cement, liberating the stereotaxic arms for holding the PV/MD optrode and the CA1/sub electrode. The optrode was assembled from a pair of stereotrodes (teflon-coated 60 μm tungsten, ~500 μm inter-pole) surrounding a stripped optical fibre (200 μm diameter, 0.22 numerical aperture) attached to a ceramic ferrule (2.5 mm diameter). Green light pulses (525 nm) were generated from a LED module (Plexon). The fibre output was checked through a photodetector kit, aiming at 6 mW power both before and after the implantation. So, the estimated irradiance^59^ at 1 mm from the tip was 1.84 mW/mm^2^.

For automatic delivery of electrical (CA1/sub) and optical pulses (PV/MD), we used the same stimulator of the main experiment. This time, it was controlled by electronic prototyping (Arduino), allowing us to customize the co-stimulation: CA1/sub paired pulses every 10 s with PV/MD light on or off, randomly. CA1/sub electrical pulses were programmed to be in the middle of PV/MD light pulses (3 s). Remaining parameters were the same as the main experiment, except for MUA (not SUA) recordings, and the absence of HFS. See Fig. 6a.

To confirm the PV/MD transfection, paraformaldehyde-fixed brains were immersed in buffered 20% sucrose overnight, and frozen in dry ice chilled-isopentane for cryostat sectioning. Coronal sections (8 μm) were placed on glass slides, and submitted to 5 min Hoechst 33342 (H1399, Molecular Probes) in PBS (4 µg/ml) to stain nuclei. Sections were then washed and coverslipped. Transfection was evaluated through fluorescence microscopy.

Due to the multiplicity of variables of this experiment, and to the fact it is complementary, we report data from two rats out of six attempts. Criteria for discarding rats were (in this order of importance): (1) detectable optical drive of PV/MD; (2) signal/noise ratio of mPFC MUA; and (3) consistency of CA1/sub-mPFC recruitment.

### Data analysis

Data were analysed through Matlab. Perievent windows were based on timestamps from the stimulator. Inconsistencies between these timestamps and the actual stimulus artefacts (~20 ms delays) were corrected subject-by-subject.

Perievent SUA analysis aimed at: (1) 1.4 s histograms (10 ms bins, 400 ms pre-event) Z-scored against the pre-event period; and (2) 180 ms histograms (3 ms bins, 20 ms pre-event). The 180 ms windows frequently showed no spikes in their pre-event periods, explaining why we did not Z-score them. In both cases, data were averaged across recording periods: final 15 min of the baseline, initial 15 min post-HFS, and last 15 min of the recording session. Spontaneous SUA, in turn, was processed as 3 min-binned Z-scored histograms. Samples of histograms were compared through two-way repeated measures ANOVA (between factor: recording period or brain site; within factor: time; alpha: 0.05).

Perievent LFP analysis aimed at 180 ms-windowed fPSP, comprising: 20 ms before the conditioning pulse, 80 ms after it (fPSP1), and 80 ms after the test pulse (fPSP2). Voltage amplitudes were then measured within the 5–14 ms latency, and averaged every 18 sweeps (i.e., 3 min). Two analyses were made thereafter: (1) fPSP1 and fPSP2 amplitudes relative to the baseline mean; and (2) paired-pulse ratios, i.e., fPSP2/fPSP1 amplitudes. Sequences of fPSP amplitudes and paired-pulse ratios were compared through two-way repeated measures ANOVA (between factor: brain site; within factor: time).

For the co-stimulation experiment, thalamic MUA was analysed in 9 s perievent histograms (50 ms bins) covering three segments: before, during, and after light (3 s each). In turn, prefrontal MUA was extracted in 1.4 s perievent windows as above. Sweeps with or without light were then sorted. Samples of histograms were compared through two-way repeated measures ANOVA (between factor: trial type, i.e., light-on *vs*. light-off; within factor: time). LFP was analysed through the multi-taper method (Chronux^60^; 1–4 Hz band-pass; 30 s windows in 2 s steps; time-bandwidth product: 3; tapers: 5). Resulting spectrograms were converted to decibels and Gaussian-smoothed.

Immunohistochemistry was analysed using published protocols^61^. Photomicrographs (mPFC, PV/MD) were captured using bright-field microscopy at 200x magnification, and constant illumination (3 V, 100% exposure). Images were converted to the 8-bit grey scale, and regions of interest were delimited with a freehand tool (ImageJ). Pixels with a grey value above an arbitrary threshold were deemed Zif268-positive, and quantified as area percentage. Percentages were then examined for Spearman’s rank correlations with electrophysiological values from the post-HFS period: (1) amplitudes of fPSP2; and (2) moduli of MUA responses. False positives were minimized through the False Discovery Rate adjustment^62^.

### Data availability

The datasets generated during and/or analysed during the current study are available from the corresponding author on reasonable request.
